# Complete Genome Sequences of Getah Virus Strains Isolated from Horses in 2016 in Japan

**DOI:** 10.1128/genomeA.00750-17

**Published:** 2017-08-03

**Authors:** Manabu Nemoto, Hiroshi Bannai, Akihiro Ochi, Hidekazu Niwa, Satoshi Murakami, Koji Tsujimura, Takashi Yamanaka, Hiroshi Kokado, Takashi Kondo

**Affiliations:** a Equine Research Institute, Japan Racing Association, Tochigi, Japan; b Thermo Fisher Scientific, Life Technologies Japan Ltd., Tokyo, Japan

## Abstract

Getah virus is mosquito-borne and causes disease in horses and pigs. We sequenced and analyzed the complete genomes of three strains isolated from horses in Ibaraki Prefecture, eastern Japan, in 2016. They were almost identical to the genomes of strains recently isolated from horses, pigs, and mosquitoes in Japan.

## GENOME ANNOUNCEMENT

Getah virus belongs to the genus *Alphavirus* in the family *Togaviridae* (1). It is a mosquito-borne virus that causes fever, skin eruptions, and limb edema in horses (1) and fetal death and reproductive disorders in pigs (2, 3). The first outbreak among horses was reported in 1978 at the Miho training center of the Japan Racing Association in Ibaraki Prefecture, eastern Japan, which usually houses about 2,000 racehorses (4). An inactivated vaccine was developed and has been used in Japan since 1979. After 1979, there were no reported outbreaks of Getah virus infection among vaccinated horses in Japan until sudden outbreaks were reported at the Miho training center in 2014 and 2015 (5, 6). An epidemic occurred at the same location in 2016. In addition, Getah viruses were isolated from mosquitoes in 2012 (7) and pigs in 2015 (8). Here, we read the complete genome sequences of the strains isolated from horses in 2016 and compared them with those of the strains recently isolated from horses, pigs, and mosquitoes.

EDTA-treated blood samples were collected from pyretic horses in 2016. Three strains (16-I-599, 16-I-674, and 16-I-676) from blood samples that were positive for Getah virus by reverse transcription-PCR (9) were successfully isolated in Vero cells (10). The genomes of the three strains were sequenced using the Ion Personal Genome Machine system (Thermo Fisher Scientific, Waltham, MA, USA), as described previously (8). The raw signal data were analyzed by using Torrent Suite version 5.0.5, and 14-I-605-C1 (GenBank accession no. LC079088) was used as a reference sequence. The 5′- and 3′-end sequences were determined by rapid amplification of cDNA ends, as described previously (8).

The complete genomes of 16-I-599, 16-I-674, and 16-I-676 were 11,690, 11,689, and 11,689 nucleotides long [excluding the 3′-poly(A) tails], respectively. The nucleotide sequence identities among the three strains isolated in 2016 ranged from 99.95% to 99.96%. The complete genomes of these three strains shared 99.89% to 99.91% nucleotide sequence identity with 14-I-605-C1 and 14-I-605-C2, isolated from a horse at the Miho training center in 2014 (11); 99.92% to 99.95% identity with 15-I-752, isolated from a horse at the same center in 2015 (5); 99.93% to 99.96% with 15-I-1105, isolated from a pig in 2015 (8); and 99.91% with 12IH26, isolated from mosquitoes in 2012 (7). Strain 15-I-1105 was isolated in the city of Tsuchiura, in Ibaraki Prefecture, which is located close to the training center (8). These results suggested that one strain had circulated among horses from 2014 to 2016 at the training center and had circulated interactively between horses and pigs (8). The three strains isolated in 2016 were also very similar to 12IH26, the strain isolated from mosquitoes in 2012 in the city of Isahaya, Nagasaki Prefecture, southwestern Japan, about 1,000 km from the training center. Mosquitoes blown on westerly winds might have transferred recent Getah virus strains from southwestern to eastern Japan.

### Accession number(s).

Strains 16-I-599 (accession no. LC223130), 16-I-674 (accession no. LC223131), and 16-I-676 (accession no. LC223132) have been deposited in the GenBank/EMBL/DDBJ databases.
